# Surface properties and initial bacterial biofilm growth on 3D-printed oral appliances: a comparative in vitro study

**DOI:** 10.1007/s00784-022-04838-7

**Published:** 2022-12-28

**Authors:** Sabina Noreen Wuersching, David Westphal, Bogna Stawarczyk, Daniel Edelhoff, Maximilian Kollmuss

**Affiliations:** 1 Department of Conservative Dentistry and Periodontology, University Hospital, LMU Munich, Goethestrasse 70, 80336 Munich, Germany; 2 Department of Prosthetic Dentistry, University Hospital, LMU Munich, Goethestrasse 70, 80336 Munich, Germany

**Keywords:** 3D printing, Occlusal splints, Bacterial adhesion, Biofilm, Surface roughness

## Abstract

**Objectives:**

To investigate the initial bacterial adhesion on 3D-printed splint materials in relation to their surface properties.

**Materials and methods:**

Specimens of five printable splint resins (SHERAprint-ortho plus UV, NextDent Ortho Rigid, LuxaPrint Ortho Plus, V-Print Splint, KeySplint Soft), one polymethylmethacrylate (PMMA) block for subtractive manufacturing (Astron CLEARsplint Disc), two conventional powder/liquid PMMA materials (FuturaGen, Astron CLEARsplint), and one polyethylene terephthalate glycol (PETG) thermoplastic sheet for vacuum forming (Erkodur Thermoforming Foil) were produced and finished. Surface roughness *R*_*a*_ was determined via contact profilometry. Surface morphology was examined under a scanning electron microscope. Multi-species bacterial biofilms were grown on entire splints. Total biofilm mass and viable bacterial counts (CFU/ml) within the biofilms were determined. Statistical analyses were performed with a one-way ANOVA, Tukey’s post hoc test, and Pearson’s test (*p* < 0.05).

**Results:**

Astron CLEARsplint and KeySplint Soft specimens showed the highest surface roughness. The mean total biofilm mass on KeySplint Soft splints was higher compared to all other materials (*p* < 0.05). Colony-forming unit per milliliter on FuturaGen, Astron CLEARsplint, and KeySplint Soft splints was one log scale higher compared to all other materials. The other four printable resins displayed overall lower *R*_*a*_, biofilm mass, and CFU/ml. A positive correlation was found between *R*_*a*_ and CFU/ml (*r* = 0.69, *p* = 0.04).

**Conclusions:**

The 3D-printed splints showed overall favorable results regarding surface roughness and bacterial adhesion. Thermoplastic materials seem to display a higher surface roughness, making them more susceptible to microbial adhesion.

**Clinical relevance:**

The development of caries and gingivitis in patients with oral appliances may be affected by the type of material.

## Introduction

Oral appliances are useful devices for treating various conditions in the maxillofacial region. In prosthetic dentistry, occlusal splints are frequently used in the management of temporomandibular disorders (TMD) as an additional treatment strategy to home training and relaxation techniques [1]. For patients suffering from sleep bruxism, it is advisable to wear a nightguard to protect the dentition from attrition caused by clenching and grinding [2]. Furthermore, oral appliances are also used in orthodontics, for example, as clear aligners for orthodontic tooth movement or as retention splint for maintaining the therapy results after the completion of the active orthodontic phase [3, 4].

Since oral appliances are typically made of resin and are intended to be worn for several hours a day, they are a potential target for bacterial adhesion and biofilm formation by the bacteria inhabiting the oral cavity. Occlusal splints used for treating TMD or sleep bruxism are predominantly worn during the night, whereas orthodontic clear aligners or retention splints may remain in the oral cavity for up to 22 h a day [5]. The accumulation of biofilm over time eventually leads to matured plaque, which is not easily washed off from the surface unless mechanically removed. Thick layers of plaque on the surface of oral appliances are a potential cause of gum irritations and may increase the risk for developing oral diseases such as caries or oral candidiasis [6–8].

Bacterial adhesion after sufficient polishing of the resin material is markedly affected by its surface properties, such as the surface topography, the surface free energy, and the surface roughness [9, 10]. It has been suggested that an increased surface roughness favors microbial adherence since it protects bacteria from shear forces during initial attachment and offers a larger area for biofilm formation [10, 11]. Since there are several methods for manufacturing resin oral appliances, these surface properties may vary depending on each method. Traditional heat-cured manufacturing is based on mixing powder and liquid at defined ratios to form a viscous resin mass, which is then poured into a mold providing the desired shape of the splint and polymerized under pressure and heat. A different approach for producing splints is vacuum-forming, where a resin sheet is heated until soft and draped over a dental cast under application of suction force. Vacuum-formed splints are widely used for orthodontic aligners but unfortunately show a low wear resistance, which makes them unsuitable for long-term treatments with occlusal splints [12].

Due to the increasing demand for digitalizing workflows in dental offices and dental laboratories, computer-aided design (CAD) and computer-aided manufacturing (CAM) technologies have become essential methods for producing resin restorations. Compared to traditional manufacturing, CAD/CAM techniques show several advantages in terms of throughput, complexity, precision, and customization [13, 14]. One of the most recent developments in CAD/CAM technology is additive manufacturing, commonly known as 3D printing, which is gaining popularity among both clinicians and dental technicians. 3D printing is based on the concept that light of a specific wavelength is directed to a vat containing a liquid photosensitive resin, which is locally cured and solidified layer by layer to create an object in the desired shape [14]. 3D printing allows the precise fabrication of complex structures and also has the advantage that it is less wasteful than subtractive manufacturing, where the specimen is milled from an entire material block [15]. However, the degree of conversion achieved in most 3D printers is often not enough for ensuring sufficient mechanical properties and thus 3D-printed objects usually require post-processing steps, such as washing off excess resin and further light curing [16, 17].

Over the past few years, several printable resins for producing 3D-printed splints have been introduced. However, there are only few data about the surface properties of 3D-printed splints. Since oral appliances are large in size and cover the entire tooth surface, it is important to obtain information on how bacterial adhesion and plaque formation is affected by different types of splint materials. Therefore, this study was conducted to assess the surface properties of occlusal splints made of five different printable resins compared to milled, vacuum-formed, and heat-cured splints. Furthermore, this study aims to examine bacterial biofilm growth on the splint surfaces using a polymicrobial biofilm model composed of bacteria that are typically found in supragingival plaque.

## Materials and methods

### Oral splint materials

Five resin-based materials for 3D-printed oral splints were examined in this study and compared to one industrial polymethylmethacrylate (PMMA) resin blank for subtractive manufacturing, two types of conventional PMMA materials based on a powder and liquid system, and one type of polyethylene terephthalate glycol (PETG) thermoplastic for vacuum forming. Table 1 shows an overview of all materials along with the abbreviations used from this point on.

**Table 1:** Splint materials used in this study

Material (Abbr.)	Manufacturer	Lot No	Manufacturing technique	Polymerization and post-processing steps as required by the manufacturer
Erkodur Thermoforming Foil 1.0 mm (ED)	Erkodent Erich Kopp GmbH (Pfalzgrafenweiler, Germany)	11150	Thermoforming	Not applicable
FuturaGen (FG)	Schütz Dental GmbH (Rosbach, Germany)	2021008951 2021003886	Conventional (powder/liquid)	Polymerization in a pressure pot (4 bar, 50 °C) for 30 min
Astron CLEARsplint (CS)	Astron Dental Corporation (Lake Zurich, IL, USA)	86562–2 E71414-10	Conventional (powder/liquid)	Polymerization in a pressure pot (4 bar, 50 °C) for 45 min
Astron CLEARsplint Disc (CD)	Astron Dental Corporation	E71406-4 /86561–5	Milling	Not applicable
SHERAprint-ortho plus UV (SP)	SHERA Werkstoff Technologie (Lemförde, Germany)	241212	3D printing	3-min ultrasonic precleaning with SHERAultra-p, 2-min ultrasonic cleaning with fresh SHERAultra-p, heating to 40 °C for 30 min, post-exposure in xenon photo flash unit with 2 × 2000 flashes under protective nitrogen gas atmosphere
NextDent Ortho Rigid (ND)	NextDent (Centurionbaan, Netherlands)	XG121N02	3D printing	3-min ultrasonic precleaning with 96% ethanol, 2-min ultrasonic cleaning with fresh 96% ethanol, 10-min UV post-exposure at 60 °C
LuxaPrint Ortho Plus (LP)	DMG (Hamburg, Germany)	217242	3D printing	Cleaning with isopropanol in *P* wash cleaning device, post-exposure (*P* cure)
V-Print Splint (VP)	Voco GmbH (Cuxhaven, Germany)	2111271	3D printing	3-min ultrasonic precleaning with isopropanol, 2-min ultrasonic cleaning with fresh isopropanol, 30-min post-exposure (*P* cure)
KeySplint Soft (KS)	Keystone Dental Group (Burlington, MA, USA)	KK6244	3D printing	3-min ultrasonic precleaning with isopropanol, 2-min ultrasonic cleaning with fresh isopropanol, 6-min post-exposure (*P* cure)

### Manufacturing and finishing of the splints

A digital oral splint was designed using a CAD software (Tizian Creativ RT-Software, Schütz Dental GmbH, Rosbach, Germany) and exported as a standard tessellation language (stl) file. The template was sent to a DLP printer (P30, RapidShape GmbH, Heimsheim, Germany), and additive manufacturing of SP, LP VP, and KS splints was conducted. ND splints were produced in a different 3D printer (NextDent 5100, NextDent, Centurionbaan, Netherlands). ED splints were thermoformed in a vacuum forming machine (Erkopress_motion, Erkodent) using a 3D-printed dental model and 1-mm thick Erkodur sheets (Erkodent). CC splints were milled from round blanks in a dental milling unit (CORiTEC 350i, imes-icore GmbH, Eiterfeld, Germany) using the same template as above. FG and CS splints were formed by combining the required amounts of powder and liquid and pouring the liquid resin into a blank mold. The PMMA polymers were polymerized in a pressure pot (4 bar, 50 °C) for 30 min (FG) or 45 min (CS), and the splints were then milled from the PMMA blanks as described above (CORiTEC 350i). The 3D printed splints were washed and post-cured according to the manufacturers’ instructions. Details on the post-processing of all splints is shown in Table 1. All splints were finished with the same method following a standard protocol which is routinely used in dental laboratories: a crosscut tungsten carbide bur was first used for surface grinding and smoothing the edges of the splint. Pre-polishing was performed in a dental polishing unit with a pumice powder and water mixture, and final high gloss polishing was achieved with a goat hair polishing brush and a high gloss polishing paste.

### Surface properties

#### Surface roughness measurement

Six cuboid-shaped specimens (10 mm × 10 mm × 5 mm) of each material were prepared and finished as described above. Surface roughness was assessed by measuring each specimen three times in three different directions using a tactile profilometer (MarSurf SD 26, Mahr GmbH, Göttingen, Germany). The three measurements performed for each specimen were used to calculate the average surface roughness (*R*_*a*_). The profilometer was calibrated with a reference which block the measurements that were taken for each group.

#### Analysis of surface morphology via scanning electron microscopy

For examining the surface morphology under a scanning electron microscope (SEM), additional specimens were cut from the molar tooth region of each splint. The specimens were then attached to stubs and sputter-coated with a 10–20-nm thick Au–Pd layer for conductivity (SC762, Quorum Technologies, Laughton, UK). The specimens were examined under a field emission SEM (Zeiss Supra 55 VP, Carl Zeiss, Oberkochen, Germany) at an accelerating voltage of 10 kV and a working distance of 7–10 mm. Images were taken at × 100 magnification.

### Bacterial biofilm growth

#### Bacterial strains and growth media

All bacterial strains were obtained from the German Collection of Microorganisms and Cell Cultures (DSMZ, Braunschweig, Germany). *Actinomyces naeslundii* (DSM 17233), *Streptococcus gordonii* (DSM 6777), *Streptococcus mutans* (DSM 20523), *Streptococcus oralis* (DSM 20627), and *Streptococcus sanguinis* (DSM 20567) were used in this study. All strains were grown and maintained on Schaedler agar plates supplemented with vitamin *K*_1_ and 5% sheep blood (Becton Dickinson, Franklin Lakes, NJ, USA). For growth in liquid media, all of the bacteria were cultured in Brain–Heart-Infusion Broth (BHI, Becton Dickinson) supplemented with hemin (5 µg/ml) and vitamin *K*_1_ (1 µg/ml). The bacteria were grown at a temperature of 37 °C and a humidity level of 60% in a CO_2_ enriched atmosphere with 5.8% CO_2_.

#### Biofilm growth on the oral splints

For each bacterial species, a sterile inoculation loop was used to scrape the colonies off the agar plates, until a pellet of biomass accumulated on the loop. *A. naeslundii*, *S. gordonii*, *S. mutans*, *S. oralis*, and *S. sanguinis* were inoculated in liquid media by transferring the biomass on the loops to individual flasks containing 60-ml BHI. The bacteria were incubated overnight and grown to their individual stationary phase, which had been determined for each species in a previous experiment (data not shown). The bacterial suspensions were diluted with fresh BHI and adjusted to an optical density (OD) yielding approximately 10^5^ bacteria [18]. Equal volumes of the five diluted suspensions were combined in a glass flask. The two fragments of each oral splint were disinfected with 100% isopropyl alcohol for 1 min and left to air-dry. The splint fragments were placed in a Falcon tube, and 40 ml of the bacterial multi-species suspension was added. The tubes were incubated for 72 h, allowing biofilm growth on the oral splints.

#### Total biofilm mass

The total biofilm mass grown on the oral splints was quantified with a crystal violet staining method. The splint fragments were rinsed in 0.9% sodium chloride to remove non-adherent cells and then stained with a 0.1% aqueous crystal violet solution and incubated for 10 min at room temperature. The specimens were removed from the crystal violet solution, and the excess staining solution was discharged by drying the specimens with a clean paper towel. The stained splints were then moved to clean tubes, and the dye was solubilized by adding 30% acetic acid and incubating the tubes on an orbital shaker at 50 rpm for 10 min at room temperature. The contents of each tube were briefly mixed by pipetting and for every specimen, two replicates containing each 100 µl of the crystal violet solution/acetic acid solution were added to the wells of an optically clear flat-bottom 96-well plate. The OD was measured at 600 nm (OD_600_) in a microplate reader (Varioskan Microplate Reader, Thermo Fisher Scientific, Waltham, MA, USA), and mean values of the two replicates were calculated for every disc.

#### Number of viable bacteria within the biofilms grown on the oral splints

The oral splints with grown multi-species biofilms were washed in 0.9% sodium chloride to remove any loose cells from the surface and stored in 30 ml of fresh sodium chloride solution for 1 h. Biofilms were extracted using a modified three step method as previously described [18, 19]. Tubes containing the splint fragments in sodium chloride solution were vortexed for 60 s, followed by 60 s of probe-based sonication at 8 W on ice, and further vortexing for 60 s. The surface of the samples was visually checked for any residual biomass prior to proceeding with the protocol. Tenfold serial dilutions of the sonicates were prepared in sodium chloride and plated on agar plates. After incubation for 48 h, the number of viable cells within the sonicates was quantified by counting the colony-forming units (CFUs) following FDA guidelines (only plates with 25–250 colonies were considered).

### Statistical analyses

All statistical analyses were implemented in Python 3.8.0 [20]. The packages *scipy* and *scikit* were used for inferential statistics, and *matplotlib* was used for the descriptive analyses. Homoscedasticity was assessed with Levene’s test, and data were tested for normality with the Shapiro–Wilk test. Comparisons between groups with parametric data were performed using a one-way analysis of variances (ANOVA) and Tukey’s post hoc analysis (*p* < 0.05). Possible associations between the surface roughness and the bacterial adhesion were assessed by calculating Pearson’s *r* coefficient at an alpha level of 0.05.

## Results

### Surface roughness

The average surface roughness (*R*_*a*_) of the tested splint materials is presented in Table 2. The highest *R*_*a*_ values were registered for CS and KS specimens. CD and FG showed the second highest surface roughness. *R*_*a*_ values of the other printable resins SP, ND, LP, and VP were in a similar range. The lowest surface roughness was found for ED. The *R*_*a*_ for all tested materials in descending order was CS > KS > CD > FG > LP > ND > VP > SP > ED.

**Table 2:** Average surface roughness *R*_*a*_ [µm] of the resin materials measured via contact profilometry. Data shown as means and standard deviation

	ED	FG	CS	CD	SP	ND	LP	VP	KS
*R*_*a*_	0.028 ± 0.011	0.200 ± 0.035	0.338 ± 0.079	0.211 ± 0.043	0.115 ± 0.026	0.164 ± 0.033	0.179 ± 0.075	0.137 ± 0.040	0.315 ± 0.108
*p* < 0.05	FG, ND, LP	ED, CS	FG, CD, LP	CS		ED, KS	ED, CS, KS		ND, LP
*p* < 0.001	CS, CD, KS		ED, SP, ND, VP	ED	CS, KS	CS		CS, KS	ED, SP, VP

Multiple comparisons were performed using ANOVA and a Tukey HSD post-hoc test

### Surface morphology

**Fig. 1:**
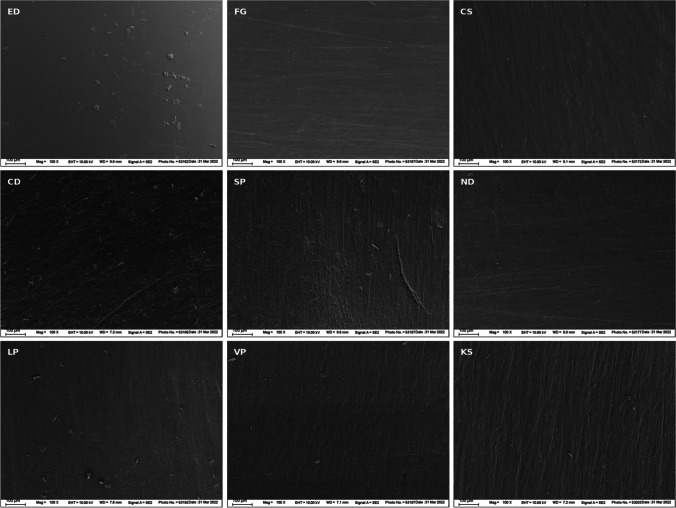
SEM images of the surfaces of the splint specimens at × 100 magnification

Sample images from the SEM analysis of the surface of the splint specimens are shown in Fig. 1 (× 100 magnification). The images reveal differences in the microstructure of the tested materials. ED showed the smoothest surface with minor scratches and irregularities. All other materials had microscopical grooves on their surface from the finishing procedure with polishing brushes, but CS and KS displayed the most pronounced grooves. Apart from minor imperfections, the printable materials SP, ND, LP, and VP showed a similar surface morphology in the SEM images.

### Bacterial biofilm growth

**Fig. 2:**
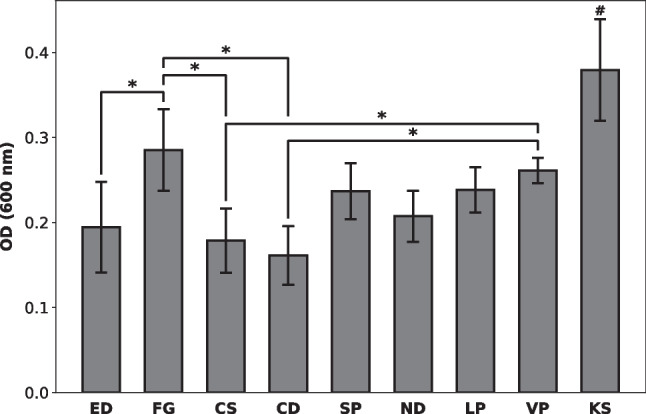
Bacterial biofilm growth on the surface of resin splints. Total biofilm mass after crystal violet staining and solubilization with 30% acetic acid. Data shown in terms of OD_600nm_. *p < 0.05; ^#^*p* < 0.05 between KS and all other test groups

**Fig. 3:**
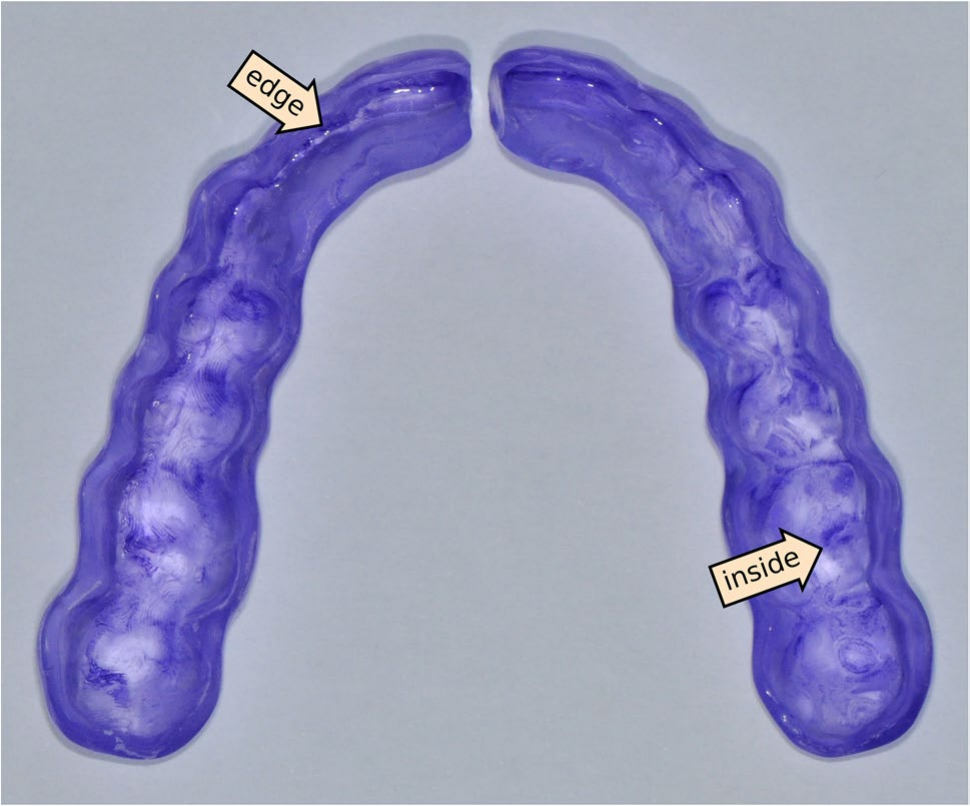
Sample image of a stained splint. Arrows are directed to exemplary areas showing that biofilm mass mainly accumulated at the edges and on the inside of the splint

The total biofilm mass grown on the tested resin splints is shown in Fig. 2. The mean total biofilm mass determined with CV staining was the highest on KS splints. Biofilm mass on all other splint materials was lower compared to KS (*p* < 0.05). The second highest biofilm mass was observed on FG splints, whereas ED, CS, and CD showed the lowest mean CV staining values. The biofilm mass detected on SP, ND, LP, and VP splints was in the same range. Regardless of the material, biofilm mass mainly accumulated on the inside and at the edges of the splints. Figure 3 shows a sample image of a splint stained with CV.

**Fig. 4:**
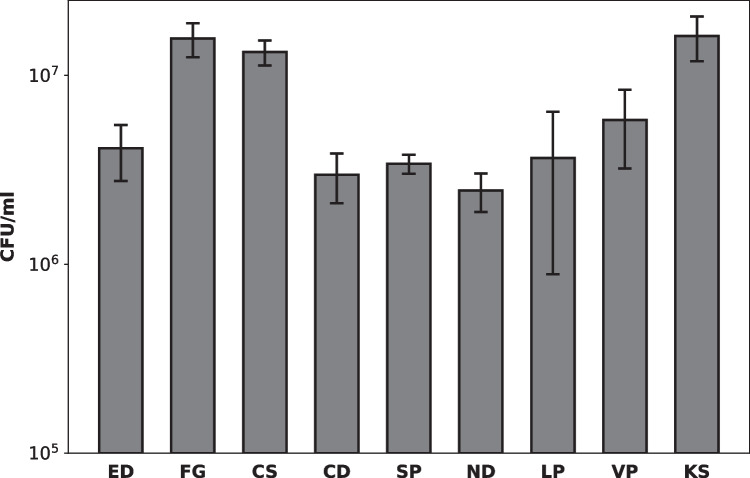
Number of viable cells determined after plating and culturing biofilm extracts grown on the splints. Data shown in terms of CFU/ml

Viable cells (CFU/ml) extracted from the biofilm sonicates are displayed in Fig. 4. The highest number of viable bacteria cultivated from the biofilm sonicates were found in biofilms grown on FG, CS, and KS splints. The CFU/ml for FG, CS, and KS was higher compared to all other materials and differed by approximately one log scale. The number of bacteria cultivated from biofilm sonicates of ED, CD, SP, ND, LP, and VP splints was in a similar range.

### Correlations between surface properties and bacterial adhesion

The association between the surface roughness and bacterial adhesion was assessed by calculating Pearson’s correlation coefficient *r* at an alpha level of 0.05. The correlation test showed a strong positive correlation between the surface roughness *R*_*a*_ and the viable bacterial count in terms of CFU/ml (*r* = 0.69, *p* = 0.04). No significant correlation was found between the surface roughness *R*_*a*_ and the total biofilm mass OD_600_.

## Discussion

Five commercially available materials for 3D printing were chosen for this study and compared to two conventional PMMA materials based on a powder and liquid system, one PMMA block for subtractive manufacturing, and one thermoforming foil. The relationship between surface properties of polymer materials and microbial adhesion has been investigated in previous studies. However, most of these studies use mono-species biofilms, often only with *S. mutans*, to investigate biofilm growth [9, 21–23]. This is a simple, but not quite realistic approach, because biofilms in the oral cavity rarely consist of just one bacterial species. Instead, oral biofilms are typically polymicrobial in nature, and their pathogenic potential varies depending on the microbial composition. With our choice of bacteria, we aimed to mimic a biofilm with cariogenic and gingivitis-inducing potential, since caries and gingivitis are the two most frequent complications during or after long-term treatments with oral appliances. As opposed to many previous studies, our biofilm model comprises five typical species found in supragingival dental plaque: the three streptococci *S. gordonii*, *S. oralis*, and *S. sanguinis* as well as *A. naeslundii* are frequent pioneer colonizers during initial biofilm formation and support the attachment of succeeding microorganisms [24]. They have been shown to interact with other bacterial species via a mechanism known as coaggregation, thereby contributing to a complex biofilm ecology [25]. By establishing an acidic environment, the early colonizing streptococci are thought to prepare the ground for the incorporation of more acidogenic bacteria into the biofilm, such as *mutans* streptococci [26]. Because of its key role in the development of dental caries, *S. mutans* was added to the multi-species biofilm model. The two methods we used for quantifying the biofilms on the splints have been previously described in the context of examining early stages of biofilm formation in vitro [19, 27, 28]. Crystal violet staining is a reliable method for determining biofilm mass, especially when gram-positive bacteria are involved. However, colorimetric staining techniques are semi-quantitative methods and are not representative of the number of viable bacteria embedded within the biofilm. This is because crystal violet not only stains bacterial cells but any material adhering to the surface, such as matrix components, and may overestimate the number of adherent bacteria on the stained surface. Therefore, we added a second method to our study to determine the CFU/ml within the biofilms extracted from the splint surfaces. The combination of vortexing and sonification in a three-step protocol has been shown to be the most efficient method for extracting and quantifying polymicrobial biofilms grown on the surface of medical devices [18].

For this study, we used entire splints for the microbiological evaluations instead of small specimens, such as discs or cubes, which are commonly used for biofilm studies on biomaterials. However, for the materials examined in this study, small specimens are not quite suitable for the following reasons: small specimens with a simple shape are easier to polish than entire splints, because the latter has areas that may not be accessible to polishing due to their more complex geometry. In fact, to keep a good fit on the tooth-splint interface, the inside of oral splints deliberately remains unpolished in clinical practice. These areas, having a rougher surface by definition, are not considered when working with small specimens that are polished on all sides and hence display an overall smoother surface. By means of the crystal violet staining protocol, we were able to judge which regions of each splint were more prone to microbial adhesion. As expected, we observed biofilm growth mainly on the inside of the splints, that is, the part which comes into direct contact with the tooth surface. Further areas with enhanced biofilm growth were the edges of the splints close to the gingiva. This was observed with all splints regardless of the material, though not to the same extent. This distribution pattern could promote gingivitis when applied to in vivo conditions, especially in view of the fact that rough margins retaining biofilm are known to promote gingiva irritation [29, 30]. Furthermore, the biofilm-coated areas on the inside of the splints close to the tooth surface may also increase the risk for caries, especially in view of the time the devices remain in the oral cavity in patients undergoing splint therapy or orthodontic treatment with aligners.

As far as the surface roughness is concerned, CS, KS, and CD showed the highest *R*_*a*_ values. SEM analysis also revealed that polishing produced more pronounced grooves on the surface of these materials compared to the other ones. One thing KS, CS, and CD have in common is that they have thermoplastic properties; that is, they become pliable upon exposure to higher temperatures. These splints can be placed in warm water at temperatures between 40 and 45 °C before inserting in the mouth, which is supposed to improve patient comfort and lead to a more precise fit. However, the user is explicitly warned to not exceed water temperatures above 45 °C, or else the device may deform. These thermoplastic features may have been responsible for an increased surface roughness, assuming that the friction generated while polishing the surface led to a temperature rise and perhaps even exceeded the critical temperature. Perhaps this led to a local temperature-dependent change in the material’s elastic modulus, causing plastic deformation on some areas of the splint surface. Another possible explanation for differences in surface roughness is the material’s hardness. Microhardness and surface roughness have been shown to correlate in dental composites; that is, materials with a higher microhardness were observed to show an increased surface roughness after polishing [31]. Furthermore, surface properties of 3D-printed materials have been shown to be markedly affected by the post-polymerization strategy. Recent investigations have demonstrated that surface roughness and surface hardness varied depending on the post printing cleaning and curing methods [32, 33].

Microbial adhesion in terms of viable bacteria was the highest on FG, CS, and KS splints’ however, the difference in CFU/ml compared to the other materials was only one log scale. From a biological point of view, this is only a minor difference considering that bacteria have an exponential growth curve. As far as the total biofilm mass is concerned, the highest OD values were recorded for biofilms grown on FG and KS splints. Analysis of the data obtained in this study revealed a strong positive correlation between surface roughness and the number of viable bacteria, and our findings are therefore also in agreement with results from previous studies [34]. The total biofilm mass produced by the bacteria did not correlate with the surface roughness. This discrepancy between CFU/ml and the stained biofilm mass suggests that the relationship between biofilm mass and the number of bacteria residing within the biofilm is not linear. Previous biofilm studies have also shown that biofilm thickness is linked to other parameters besides the number of bacteria involved in biofilm formation. Specifically, environmental factors such as flow, nutrient conditions, and temperature are known to influence the architecture of the biofilm matrix [35]. Perhaps the composition of the splint material was a further variable influencing the bacterial metabolism during biofilm formation and the proportion of the biofilm matrix produced by the bacteria. Another possible explanation for this discrepancy is the metabolic downshift of bacteria residing within the biofilm, a state often referred to as metabolic “dormancy” [36]. Although the bacterial metabolism is assumed to be reactivated as soon as the bacteria are released from the biofilm, we cannot be certain about metabolic state of the bacteria in the sonicates and their ability to proliferate after plating and culturing the sonicates on the agar plates.

Furthermore, it has been previously suggested that surface roughness only has a significant impact on biofilm formation if the surface has a minimum “threshold” *R*_*a*_ value of 0.2 µm [34, 37, 38]. In our case, KS, CS, CD, and FG were the only materials meeting this criterion. It seems as though this postulated relationship applies to all materials but CD, where the number of viable cells within the biofilm was lower compared to the conventionally manufactured splint CS despite having a similar surface roughness. This finding indicates that there are further parameters affecting microbial adhesion besides surface roughness and surface topography. Protein adsorption is known to be modified by factors such as surface free energy, charge, and polarity [39]. Specifically, high surface free energy, charged surfaces, and slightly hydrophilic surfaces have been shown to enhance protein adhesion [40]. The discrepancy between CS and CD suggests that the different polymerization techniques may have impacted the surface properties. CS splints were manufactured by pouring the liquid resin mass into a blank mold, which, after polymerization in a conventional pressure pot, was then milled into its final splint shape. The quality of polymerization within the depths of the CS blank may not be as good as in CD PMMA blocks, which are industrially polymerized at high temperatures and under high pressure, a procedure often referred to as HT-HP polymerization. According to the manufacturer and to data from previous studies, thermopolymerization under high pressure leads to a more homogeneous material containing fewer pores and irregularities as well as to an enhanced degree of conversion [41, 42]. Therefore, the enhanced bacterial growth on CS splints may be explained by their likely more polar and hydrophilic surface due to a higher number of unreacted monomers. It has been demonstrated that intrinsically hydrophilic surfaces have a smaller water contact angle, which facilitates surface coating of aqueous solutions, in our case, the bacterial suspension [39, 43]. Further factors influencing the initial bacterial adhesion are the pH and ionic strength of the medium as well as the presence of shear forces [39, 44]. The latter, however, probably plays a minor role in our in vitro biofilm model but has been shown to have a significant impact in vivo, where there are intraoral shear forces generated through the muscles, tongue, and salivary flow [45].

It should be noted that the significance of our results is limited by the in vitro character of our experiments, which fail to entirely replicate the complex environment in the human oral cavity. The surface properties of resin splints may be altered under in vivo conditions, for example, due to water sorption, mechanical degradation, and aging. Wear of the splints caused by mastication is an important parameter which can significantly influence the surface properties and may alter the material’s susceptibility to microbial adhesion. Therefore, in situ investigations involving splints that have been worn for a certain period of time are necessary to give us more insight into long-term biofilm growth of the materials tested in this study. For judging the clinical applicability of oral appliances, further mechanical properties such as flexural strength, hardness, and fracture toughness must be taken into account. Furthermore, it is important to obtain information about the biocompatibility of 3D-printed resin splints, especially in view of their large size. As with all polymer-based materials, there is a chance for residual monomers to leach out into the oral cavity and cause adverse effects to the surrounding oral tissues. A further limitation is the polishing regimen used in this study. We deliberately chose the same finishing procedure for all materials not only for comparability of our results but also to stay true to clinical practice, since polishing with water and pumice and a high gloss polishing paste is a standard protocol for splints routinely performed by dental technicians. Nonetheless, different polishing utensils may be required for each individual material to achieve the best surface properties. Therefore, assessing the effect of different brush types and polishing pastes on the surface parameters of resin materials may be of interest to future studies.

## Conclusions

Understanding the relationship between surface properties and bacterial adhesion is essential for preventing caries and gingivitis in patients with oral appliances. The results of our study indicate that there is a proportional relationship between surface roughness and the number of viable bacteria residing within the biofilm. Furthermore, splint materials with thermoplastic properties seem to display a higher surface roughness and more microgrooves after polishing. In conclusion, printable splint materials appear to be a good alternative to conventional powder/liquid-based resins and PMMA blocks as far as surface properties and bacterial biofilm growth are concerned. Nonetheless, further studies investigating their mechanical properties, biocompatibility, and their clinical behavior are necessary to judge their applicability in the oral cavity.

## Data Availability

Data available on request from the authors.
